# Protein Disulfide Isomerases Regulate IgE-Mediated Mast Cell Responses and Their Inhibition Confers Protective Effects During Food Allergy

**DOI:** 10.3389/fimmu.2020.606837

**Published:** 2020-12-22

**Authors:** Dylan Krajewski, Stephanie H. Polukort, Justine Gelzinis, Jeffrey Rovatti, Edwin Kaczenski, Christine Galinski, Megan Pantos, Nickul N. Shah, Sallie S. Schneider, Daniel R. Kennedy, Clinton B. Mathias

**Affiliations:** ^1^ Department of Pharmaceutical and Administrative Sciences, College of Pharmacy and Health Sciences, Western New England University, Springfield, MA, United States; ^2^ Pioneer Valley Life Sciences Institute, Baystate Medical Center, Springfield, MA, United States; ^3^ Department of Veterinary and Animal Sciences, University of Massachusetts at Amherst, Amherst, MA, United States

**Keywords:** protein disulfide isomerase, mast cells, food allergy, PDI, propynoic acid carbamoyl methyl amide

## Abstract

The thiol isomerase, protein disulfide isomerase (PDI), plays important intracellular roles during protein folding, maintaining cellular function and viability. Recent studies suggest novel roles for extracellular cell surface PDI in enhancing cellular activation and promoting their function. Moreover, a number of food-derived substances have been shown to regulate cellular PDI activity and alter disease progression. We hypothesized that PDI may have similar roles during mast cell-mediated allergic responses and examined its effects on IgE-induced mast cell activity during cell culture and food allergy. Mast cells were activated *via* IgE and antigen and the effects of PDI inhibition on mast cell activation were assessed. The effects of PDI blockade *in vivo* were examined by treating mice with the irreversible PDI inhibitor, PACMA-31, in an ovalbumin-induced model of food allergy. The role of dietary PDI modulators was investigated using various dietary compounds including curcumin and quercetin-3-rutinoside (rutin). PDI expression was observed on resting mast cell surfaces, intracellularly, and in the intestines of allergic mice. Furthermore, enhanced secretion of extracellular PDI was observed on mast cell membranes during IgE and antigen activation. Insulin turbidimetric assays demonstrated that curcumin is a potent PDI inhibitor and pre-treatment of mast cells with curcumin or established PDI inhibitors such as bacitracin, rutin or PACMA-31, resulted in the suppression of IgE-mediated activation and the secretion of various cytokines. This was accompanied by decreased mast cell proliferation, FcεRI expression, and mast cell degranulation. Similarly, treatment of allergic BALB/c mice with PACMA-31 attenuated the development of food allergy resulting in decreased allergic diarrhea, mast cell activation, and fewer intestinal mast cells. The production of T_H_2-specific cytokines was also suppressed. Our observations suggest that PDI catalytic activity is essential in the regulation of mast cell activation, and that its blockade may benefit patients with allergic inflammation.

## Introduction

IgE-mediated mast cell activation is a critical component in the induction of allergic responses to food-derived antigens (1–3). The cross-linking of food-specific antigens by IgE-bearing mast cells in the intestinal tract induces a stepwise cascade of activation-induced events, resulting first in the release of various pre-formed mediators from mast cell granules, followed by the synthesis of several *de novo* substances including various cytokines and lipid mediators. These events are tightly orchestrated involving several phosphorylative reactions that culminate in the activation of transcription factors which regulate gene expression.

We have previously shown that food-derived components such as curcumin can attenuate the development of mast cell responses during food allergy (4, 5). Curcumin, a natural product found in the spice turmeric has well-known pharmacological properties, including anti-allergic (4, 6, 7), anti-inflammatory (8), and anti-cancer activities (9, 10). Despite the interest in curcumin and its analogues as potential therapeutics (9), there is no consensus on the molecular mechanisms by which it exerts pharmacological action. While some studies have demonstrated curcumin acts upon various transcription factors to regulate the expression of enzymes and cytokines (4, 11), other studies suggest that curcumin exerts these effects by modulating the redox status of the target cell (12).

We hypothesized that dietary components such as curcumin may modulate the mast cell response during food allergy by inhibiting the direct activation of circulating proteins and enzymes and explored likely targets. One common mechanism of protein activation is through an allosteric disulfide bond, where a disulfide will rearrange to alter the intra- or intermolecular structure of the protein to activate or inactivate it (13). The rearrangement of these disulfide bonds is often accomplished through a thiol reductase enzyme such as protein disulfide isomerase (PDI).

Thiol isomerases such as PDI catalyze the breakage, formation and rearrangement of disulfide bonds, regulating protein folding within the endoplasmic reticulum (ER) (14, 15). However more recent studies have determined extracellular roles for PDI including the activation of thrombus formation (16), entry of HIV into lymphocytes (17), and the survival and progression of various cancers (18).

In this study, we verify the PDI inhibitory activity of curcumin and explore the role that PDI plays in the development of mast cell-mediated responses during food allergy using known PDI inhibitors. To date, with the exception of a postulation that PDI catalyzes the formation of IgG4 under conditions of chronic antigen exposure (19), there has been no known role of the enzyme in the development of mast cell-dependent allergic responses. Here, we show for the first time that mast cells express extracellular PDI on their surface and that blockade of PDI in mast cells suppresses their function and attenuates the development of mast cell responses during food allergy. Pretreatment of bone marrow-derived mast cells (BMMCs) with PDI inhibitors including dietary PDI modulators suppressed their activation and degranulation, resulting in the decreased expression and secretion of various mast cell-derived cytokines. Furthermore, treatment of wild-type mice with PACMA-31 (an orally active irreversible PDI inhibitor) in a model of ovalbumin (OVA)-induced food allergy resulted in a significant attenuation in the development of food allergy symptoms including decreases in allergic diarrhea, mast cell activation and allergen-specific IgE. These data demonstrate that PDI plays vital roles during mast cell-mediated responses by regulating mast cell activation and cytokine production. Furthermore, dietary components can modulate mast cell activation during allergic responses by regulating PDI activity, suggesting that blocking PDI function may prove to be of therapeutic benefit in allergic patients.

## Materials and Methods

### Animals

BALB/c mice were purchased from Taconic Farms and Envigo. All mice were bred in our facility and all animal research was approved by the IACUC of Western New England University.

### Insulin Turbidity Assay

PDI activity was measured *via* PDI-catalyzed reduction of insulin in the insulin turbidity assay as previously described by us and others (20–22). Briefly, the reaction mixture consisted of 100 mM potassium phosphate (pH 7.4), 0.75 mM DTT, 2 mM EDTA, 35 µg/ul of bovine insulin, and 0.8 µg purified human PDI in a total volume of 30 μl in a 384-well plate. The progress of the reaction was monitored for 90 min at 37°C. Curcumin, PACMA-31 or control buffer was added prior to the addition of enzyme at the concentrations indicated. PDI activity in the presence of compound was determined by the following formula: PDI activity (%) = (OD[_compound + PDI + DTT_] – OD[_DTT_])/(OD[_PDI + DTT_] – OD[_DTT_]) × 100%. Enzyme inhibition was determined by the following formula: enzyme inhibition = (1 – [OD_max{compound + enzyme}_/OD_max{buffer control + enzyme}_]).

### BMMC Culture

BMMCs were generated from naïve BALB/c mice and cultured with 10 ng/ml of rIL-3 (Shenandoah) and rSCF (Shenandoah) for >4 weeks as we have previously described (23). Harvested BMMC were positive for c-Kit and FcϵRI.

### BMMC Activation and Pre-Treatment With PDI Inhibitors

1 × 10^6^ BMMCs/ml were cultured in triplicates with 10 ng/ml IL-3 and SCF. Cells were activated by pre-sensitizing with 1 µg/ml DNP-IgE (clone SPE7, Sigma) or vehicle (medium), followed by treatment with 200 ng/ml DNP-BSA (5, 24). Increasing concentrations of curcumin (Sigma), bacitracin (Sigma), quercetin-3-rutinoside hydrate or rutin (Sigma), and propynoic acid carbamoyl methyl amide (PACMA)-31 (Sigma) or vehicle (DMSO) were added to various experimental groups for different time periods (including 30 min and 24 h) prior to challenge with DNP-BSA. Expression of cytokine genes was assessed by RT-PCR between 30 min to 1 h following activation. Assessment of secreted cytokines was performed 6 to 24 h later.

### Measurement of ER Stress

ER stress was induced by adding brefeldin A (1 µg/ml) to unactivated BMMCs or cells activated with IgE and antigen as described above. The effects of PDI inhibition were examined by treating with PDI inhibitors such as curcumin or PACMA-31. Six hours after activation with IgE/Ag, cells were stained for surface antigens such as c-Kit or FcεRI with fluorescently-labeled mAbs. Intracellular cytokine staining was then performed using a kit from Biolegend (San Diego, CA). Cells were fixed, then permeabilized and stained for various cytokines using mAbs. Cytokine-producing cells were enumerated by flow cytometry.

### Quantitative PCR Analysis and ELISAs

Quantitative RT-PCR was performed as previously described using Taqman probes (5, 24). Expression of cytokine genes (IL-4, IL-5, IL-6, IL-9, IL-10, IL-13, IL-33, TNF-α, IFN-γ) and PDI (P4HB, PDIA3) was calculated relative to GAPDH transcripts. ELISAs for mMCP-1 (Affymetrix), IL-4, IL-5, IL-6, TNF-α, and IFN-γ (Biolegend), IL-13 (R&D Systems), and OVA-IgE were performed according to manufacturers’ protocols as previously described (5, 24).

### β-Hexosaminidase (β-Hex) Assay

BMMCs were activated with DNP-IgE and DNP-BSA in the presence or absence of various PDI inhibitors. β-hex activity in cell culture supernatants was assessed as previously described by us (5, 24). Percent cellular content was calculated according to the following formula: (amount released into supernatant)/(amount in supernatant + amount in lysate) × 100.

### BMMC Proliferation

BMMCs were cultured with rIL-3 and rSCF as described above. Some groups of cells were treated with various concentrations of bacitracin, rutin or PACMA-31. Cells were counted daily for 4 to 5 days, and live cells were enumerated on the basis of trypan blue exclusion.

### Flow Cytometry

BMMCs and MC/9 cells (a murine mast cell line) were incubated with mAbs against mouse c-Kit (Biolegend), FcεRI (Biolegend), PDI (Life Technologies), and isotype controls for PDI (IgG2a/Life Technologies). Expression of cell surface PDI was assessed at various times in cells after activation with IgE and antigen and in unactivated controls. Intracellular PDI expression was assessed 30 min later in fixed and permeabilized cells. Flow cytometric analysis was performed using an Accuri C6 flow cytometer and Flow jo software.

### Western Blot

BMMCs and MC/9 cells were activated with DNP-IgE and DNP-BSA and total protein extracts were harvested after 4 h. Western blot was performed as previously described (4). PDI detection was performed using a rabbit anti-PDI mAb (1:1000; Cell Signaling).

### Food Allergy Regimen

To induce food allergy, BALB/c mice were *i.p.* immunized with 50 μg chicken egg OVA in 1 mg alum twice as previously described (5, 24). Mice were challenged *i.g.* with 50 mg OVA on 6 alternating days. Control animals were *i.p.* sensitized but not challenged with OVA. One hour after the sixth challenge, mice were sacrificed and assessed as previously described (5, 24, 25). To assess the effects of PACMA-31 exposure in allergic mice, some groups of mice (both controls and OVA-challenged animals) were gavaged with 10 mg/kg (300 µg) of PACMA-31 suspended in 250 µl 1% carboxy methyl cellulose (CMC) as previously described (4). Treatment with PACMA-31 was initiated one day prior to challenge with OVA and continued daily until sacrifice. Mice were sacrificed 1 h after the sixth challenge with OVA and food allergy parameters were assessed as previously described (4, 5, 24). The development of intestinal anaphylaxis was assessed as described below. Blood was collected for evaluation of antibodies and mMCP-1 in serum. Jejunum was collected for histological assessment of mast cells and evaluation of cytokine gene expression by RT-PCR as described above. Spleens were collected for evaluation of systemic cytokine production by T cells.

### Measurement of Intestinal Anaphylaxis

Intestinal anaphylaxis was assessed in challenged mice by scoring the percentage of animals exhibiting allergic diarrhea for one h after OVA challenge (4, 25).

### Histological Analysis and Enumeration of Mast Cells

Intestinal mast cells were enumerated as previously described by us (26). Tissue sections were stained with chloroacetate esterase (CAE) and mast cells were counted in complete cross-sections of jejunum.

### Spleen Stimulation

Spleen cells were cultured with medium, 200 μg/ml OVA or anti-CD3 and anti-CD28 and cytokines were enumerated in supernatants as previously described (5, 24).

### Statistical Analysis

Data are expressed as mean ± SEM, unless stated otherwise. Statistical significance comparing different sets of mice (between 2 groups) was determined by the unpaired Student’s t-test, whenever applicable. In experiments comparing multiple experimental groups or time points, one or two-way analysis of variance was performed followed by the Dunnett test for multiple comparisons.

## Results

### Curcumin Inhibits Protein Disulfide Isomerase Activity and Suppresses IgE-Mediated Mast Cell Activation

**Figure 1:**
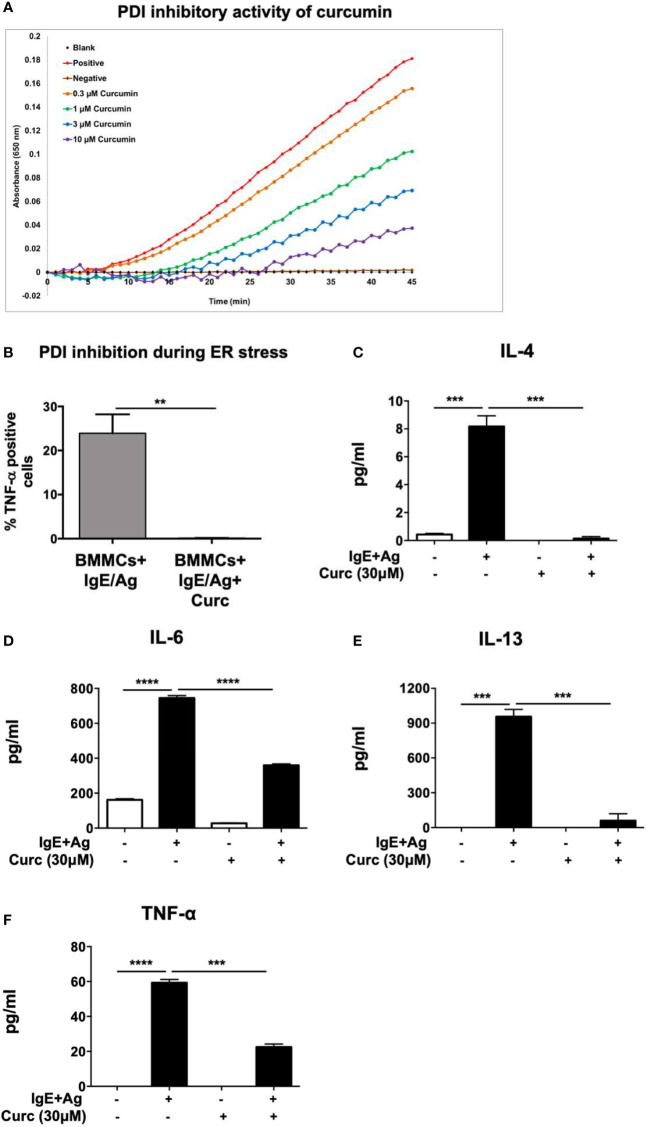
Curcumin inhibits PDI catalytic activity and suppresses IgE-mediated mast cell-derived cytokine production. **(A)** PDI activity was measured using the insulin-based turbidimetric assay in the absence (*red*) or presence of 0.3 µM (orange), 1 µM (green), 3 µM (blue), or 10 µM (purple) curcumin. The brown line represents baseline with DTT in the absence of PDI. **(B)** BMMCs were pre-treated with 30µM curcumin overnight and activated with DNP-IgE (1µg/ml) and DNP-BSA (200 ng/ml) in the presence or absence of Brefeldin A (1 µg/ml). Six h later, TNF-α-positive cells were enumerated using intracellular staining. **(C–F)** BMMCs were pre-treated with 30 µM curcumin and activated *via* DNP-IgE and antigen. Supernatants were collected 12 h later and were evaluated for the presence of IL-4, IL-6, IL-13, and TNF-α by ELISA. Data are representative of three or more independent experiments. ** p < 0.0051; ***p < 0.0005; ****p < 0.0001 (students t-test).

We have previously demonstrated that food-derived substances such as curcumin can modulate mast cell responses during food allergy by suppressing their activation and pro-allergic effects (4). The proper folding and assembly of proteins catalyzed by PDI as well as their dimerization is a critical component of cellular function, suggesting that the modulation of mast cell responses during allergic inflammation may depend on cell-specific regulation of PDI activity. Furthermore, a number of recent studies suggest novel roles for dietary PDI inhibitors in regulating cellular activation and altering disease progression (27, 28). This includes polyphenolic flavonoids such as the quercetin-3-glycosides, which have been shown to inhibit PDI both *in vitro* and in mice (20). We therefore wondered whether the effects of curcumin on mast cells may also be mediated *via* inhibition of PDI catalytic activity. Using a modified version of the insulin turbidimetric assay that measures PDI catalytic activity (20–22), we therefore explored the potential of curcumin to also inhibit PDI activity (**Figure 1A**). Curcumin inhibited PDI activity in a dose-dependent manner, suggesting that its ability to inhibit mast cell activation *in vivo* may be dependent on regulation of PDI activity (**Figure 1A**). PDI catalytic activity is highly induced during conditions of ER stress and upregulation of the unfolded protein response (UPR). To therefore further assess whether the inhibitory effects of curcumin on mast cells maybe mediated *via* PDI inhibition, we examined its role in IgE-activated mast cells under conditions of ER stress. One way of inducing ER stress in cells is to block protein transport using the chemical, brefeldin A (29). Brefeldin A inhibits transport of proteins from the ER to the Golgi and induces retrograde protein transport from the Golgi apparatus to the ER. This results in the accumulation of unfolded proteins in the ER. Examination of TNF-α cytokine production by BMMCs after IgE-induced activation using intracellular cytokine staining revealed a significant decrease in the generation of TNF-α-producing cells in the presence of curcumin (**Figure 1B**). Furthermore, pretreatment of BMMCs with curcumin prior to activation with IgE and antigen resulted in a significant decrease in the production of the cytokines IL-4, IL-6, IL-13, and TNF-α (**Figures 1C–F**). Collectively, these data demonstrate that curcumin inhibits PDI activity and its effects on mast cells may be mediated *via* PDI inhibition, warranting further assessment of the role of PDI during mast cell-mediated responses.

### Bacitracin Pre-Treatment Modulates Cytokine Gene Expression and Secretion in IgE-Activated Mast Cells

The ubiquitous expression of PDI and its importance in protein folding presents a major challenge to examination of its activity using gene knockdown strategies. Knockdown of PDI is lethal in yeast and mammalian cell lines (30) and to date, no viable strains of PDI knockout mice exist. As such, the functions of PDI both *in vitro* and *in vivo* have often been studied using various small molecule inhibitors of PDI as well as PDI-blocking antibodies. To further investigate the role of PDI during mast cell activation and function, we therefore assessed the effects of pre-treatment with various PDI inhibitors on mast cell cytokine production after activation with IgE and antigen. We explored the effects of three well-established PDI inhibitors to verify they all had the same effect and corroborate that the process was PDI-dependent. The following stepwise approach was utilized: assessment of mast cell function using classic PDI inhibitors such as bacitracin, examination of the effects of clinically validated PDI inhibitors such as rutin on mast cells, and confirmation of PDI activity in mast cells using selective PDI inhibitors such as PACMA-31.

**Figure 2:**
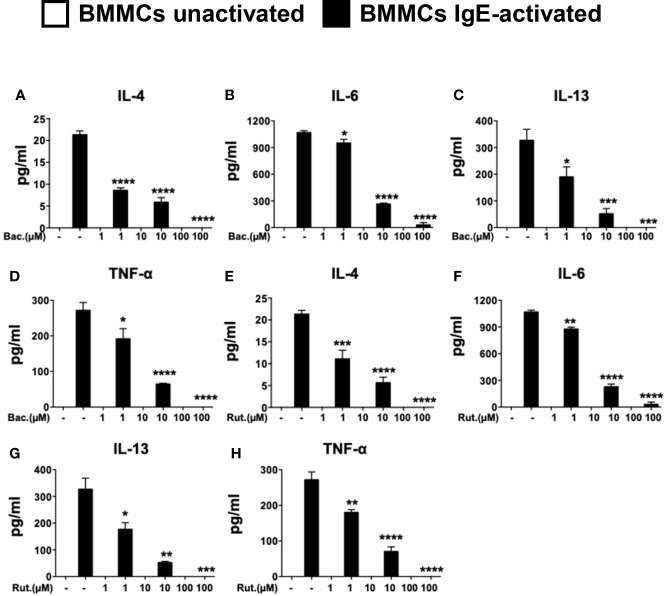
Pre-treatment with PDI inhibitors suppresses the secretion of cytokines in IgE-activated BMMCs. BMMCs were treated with increasing doses of **(A–D)** bacitracin (Bac) or **(E–H)** rutin (Rut) overnight and activated *via* IgE and antigen stimulation as in **Figure 1**. Supernatants were collected 12 h after activation and ELISAs were performed for IL-4, IL-6, IL-13, and TNF-α. Data are representative of two or more independent experiments. Statistical signifance between multiple groups was performed using one-way ANOVA with p < 0.0001. Significance for means of groups treated with Bac or Rut compared to mean of untreated cells is shown above bars representing the groups respectively. *p < 0.03; **p < 0.002; ***p < 0.0002; ****p < 0.0001 (Dunnet's post-hoc test).

The effects of PDI in cell studies have historically been studied using the topical peptide antibiotic, bacitracin (27, 31). Bacitracin is a well-studied, non-selective inhibitor of PDI and blocks PDI function in the high micromolar range (IC_50_ of 70µM in insulin reductase assays) inhibiting disulfide bond formation (32). Bacitracin is also non cell-permeable (27). Therefore, its effects on PDI catalytic activity maybe mediated *via* inhibition of cell surface PDI. To examine the effects of bacitracin pre-treatment on IgE-mediated activation in mast cells, BMMCs were pre-treated with increasing concentrations of the drug overnight prior to activation *via* IgE and antigen. The dose-dependent effects of bacitracin pre-treatment on cytokine were then assessed. As expected, IgE-mediated activation of BMMCs resulted in the enhanced secretion of IL-4, IL-6, IL-13, and TNF-α into cell culture supernatants (**Figures 2A–D**). In contrast, pre-treatment with various doses of bacitracin, resulted in a dose-dependent inhibition of cytokine secretion (**Figures 2A–D**), suggesting that bacitracin suppresses mast cell-mediated cytokine production by inhibiting the activity of PDI during IgE-induced activation. Similar effects were also observed on the induction of cytokine gene transcription (**Supplementary Figures 1A–C**). These data therefore suggest that inhibition of PDI activity can suppress the production of cytokines by IgE-activated mast cells. Furthermore, they also strongly implicate a role for extracellular PDI activity, as bacitracin is a non-cell membrane permeable PDI inhibitor.

### Rutin Pre-Treatment Modulates Cytokine Secretion in IgE-Activated Mast Cells

While bacitracin is widely used in research as a PDI antagonist, its clinical use *in vivo* is hampered by its low membrane permeability and adverse side effects such as nephrotoxicity (27). To therefore further confirm that PDI plays an important role in mast cell activation, we utilized a second well-established inhibitor of PDI, rutin (IC_50_ 6 µM in insulin reductase assays (27)), in human studies (28, 33, 34). The polyphenolic flavonoid, quercitin-3-rutinoside (rutin) was recently shown to be a potent small molecule inhibitor of PDI (20). Quercetin and its derivatives such as rutin are ubiquitously present in many fruits and vegetables (27), suggesting that they may have the potential to modulate mast cell function in a manner similar to that observed with curcumin (4). To therefore further investigate the importance of PDI during mast cell activation and confirm the effects observed above with bacitracin, we also cultured BMMCs with increasing concentrations of rutin, and assessed the production of mast cell-derived cytokines. As observed above, pre-treatment with increasing concentrations of rutin, also suppressed cytokine gene expression (**Supplementary Figures 1D–F**) and the production of mast cell-derived cytokines in a dose-dependent manner (**Figures 2E–H**), suggesting that mast cell activity during immune responses *in vivo* may be modulated by the fine-tuning of cell-specific PDI activity.

### Pre-Treatment With Bacitracin or Rutin Suppresses Release of Pre-Formed Mediators and Influences Long-Term Cell Survival

**Figure 3:**
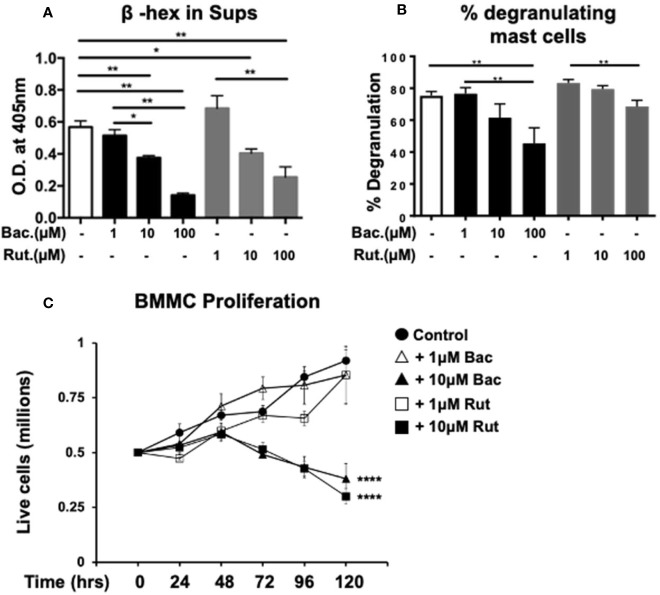
Pre-treatment with PDI inhibitors suppresses mast cell degranulation and cell proliferation. **(A, B)** BMMCs were cultured with varying concentrations of bacitracin or rutin as described above. Mast cell degranulation was assessed by pre-sensitizing BMMCs with DNP-IgE followed by challenge with DNP-BSA. Supernatants and cell lysates were collected and the β-hex assay was performed. **(A)** O.D. values correlating to β-hex release in supernatants and **(B)** percent degranulation (percent cellular content) of BMMCs is shown. **(C)** The effects of treatment with PDI inhibitors on BMMC proliferation and survival were assessed. BMMCs were cultured in triplicates with rIL-3 and rSCF for 5 days in the presence of varying concentrations of bacitracin or rutin and vehicle (DMSO). Cells were counted daily. Numbers of live cells are shown. Data are representative of 2 independent experiments. *p < 0.05; **p < 0.005 (students t-test). ****p < 0.0001 using two-way ANOVA between untreated controls and 10µM-treated groups.

Our observations above demonstrating a dose-dependent suppression of cytokine expression and secretion by PDI inhibitors are consistent with well-described roles of PDIs as molecular chaperones during the intracellular generation of various proteins. To further determine whether PDI is also involved during mast cell degranulation and the release of pre-formed mediators, we examined cell culture supernatants immediately after activation with DNP-BSA, and assessed the secretion of the enzyme β-hexosaminidase (β-hex) as previously described (24). As observed in **Figure 3A**, IgE-mediated activation induced the secretion of β-hex into cell culture supernatants 15 min later compared with unactivated controls (**Figure 3A**). In contrast, pre-treatment with increasing concentrations of either bacitracin or rutin, resulted in decreased secretion of this enzyme in activated cells (**Figure 3A**). These data suggest that inhibition of PDI can suppress the release of pre-formed mediators from mast cell granules after IgE-mediated activation. Furthermore, the total cellular β-hex content was also decreased in cells treated with PDI inhibitors (data not shown), suggesting that PDI inhibition may also block β-hex production. However, a much smaller effect on the extent of intrinsic mast cell degranulation was observed (**Figure 3B**).

To further investigate the effects of PDI inhibition on mast cells, we also examined the effects of bacitracin and rutin exposure on BMMC proliferation and survival as previously described (24). BMMCs were cultured with IL-3 and SCF, along with various concentrations of bacitracin, rutin, or vehicle. Their proliferation and/or survival was followed for 5 days and live cells were enumerated using trypan blue exclusion. As observed in **Figure 3C** and data not shown, co-culture with bacitracin or rutin for 5 days, resulted in an overall dose-dependent decrease in BMMC proliferation and survival, suggesting that PDI activity may regulate mast cell homeostasis and proliferation possibly by modulating intracellular protein folding during cell growth. This is consistent with the known roles of PDI as an important housekeeping protein necessary for cell survival (30). However, treatment with both PDI inhibitors had no effects on cell viability and proliferation for at least up to 48 h (for the 10µM doses) or longer (for the 1µM doses) after treatment. At these time points, percentages of live cells were comparable between groups and no changes in percentages of dead cells, approximately 10%, were observed. This confirms that the effects of the PDI inhibitors on IgE-mediated mast cell activation described above are not due to cell death but reduction of catalytic activity consistent with reported observations in other (27).

### Suppression of Mast Cell Activation and Function by the PDI-Selective Inhibitor, PACMA-31

**Figure 4:**
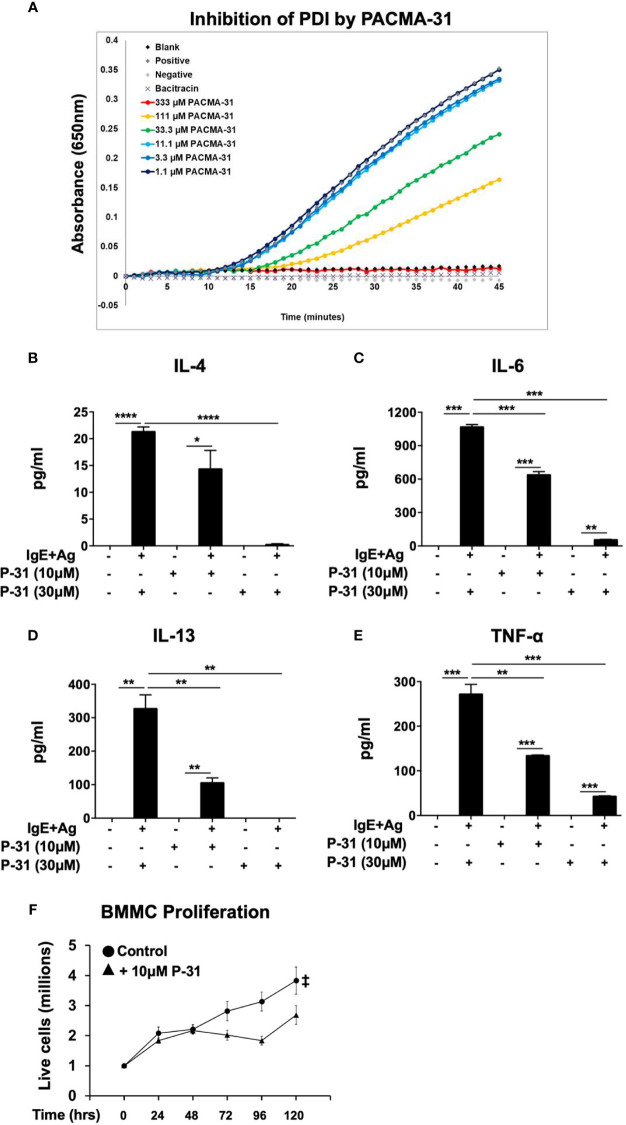
PACMA-31 inhibits PDI and pre-treatment with PACMA-31 suppresses BMMC proliferation and cytokine secretion in IgE-activated mast cells. **(A)** PDI catalytic activity was measured using the insulin-based turbidimetric assay in the absence or presence of various concentrations of PACMA-31. **(B–E)** BMMCs were treated with 10µM or 30µM PACMA-31 (P-31) overnight and activated via IgE and antigen stimulation. Supernatants were collected 12 h after activation and ELISAs were performed for respective cytokines. **(F)** BMMCs were cultured with rIL-3 and rSCF for 5 days and the effects of PACMA-31 treatment on mast cell proliferation were assessed. Numbers of live cells at different time points are shown. Data are representative of 3 or more independent experiments. *p < 0.05; **p < 0.005; ***p < 0.001; ****p < 0.0001 (student’s t-test). ^‡^p < 0.0001 by two-way ANOVA between untreated cells and P-31–treated groups.

Lastly, to verify that PDI plays an important role in these processes, we used a PDI selective compound, shown to have potent anti-PDI activity in animal studies. Recently, a class of propynoic acid carbomyl methyl amides (PACMAs) was shown to have broad-spectrum activity against various cancer cell lines (18, 35). Of these, the small molecule compound, PACMA-31, was shown to have irreversible activity against PDI (IC_50_ of 10µM in animal studies (35)), forming a covalent bond with active site cysteines, and exhibited *in vivo* activity with oral bioavailability in a mouse xenograft model of ovarian cancer (35). Furthermore, PACMA-31 has a greater selectivity for PDI over other thiol isomerases compared to bacitracin. We therefore investigated whether PACMA-31 would also inhibit PDI in our confirmatory assays and assessed its effects on mast cell activation and function in our model. As observed in **Figure 4A**, PACMA-31 is a potent inhibitor of PDI in cell-free enzymatic assays and inhibits the insulin reduction catalytic activity of PDI in a dose-dependent manner. A comparison of the PDI inhibitory activity of PACMA-31 with curcumin is shown in **Supplementary Figure 2**. To further investigate whether PACMA-31 has similar dose-dependent effects on functional studies in mast cells, we treated BMMCs with various concentrations (10µM and 30µM) of PACMA-31. As observed in the case of bacitracin and rutin above, pre-treatment of BMMCs with PACMA-31 overnight (**Figures 4B–E**) resulted in a significant suppression of mast cell activation as evidenced by reduced mast cell cytokine production (**Figures 4B–D**) and decreased overall mast cell survival (**Figure 4E**).

### Enhanced Secretion of PDI in Activated Mast Cells

Our data above demonstrates a significant reduction in mast cell functional activity and activation-induced events such as cytokine production when exposed to PDI inhibitors, suggesting that PDI enzymatic activity is induced in mast cells after activation has occurred. This is consistent with the known effects of PDI during cellular activation in various cells. Interestingly, several recent studies have also identified novel functional roles for *extracellular* secreted PDI in various cells (16, 36–40). PDI bound to integrins on the surfaces of these cells has been shown to exert extracellular effects and modulate cellular function. As such, we wondered whether extracellular PDI is also secreted by mast cells during IgE-activation and whether cell-surface PDI may likely be a target of our PDI inhibitors.

**Figure 5:**
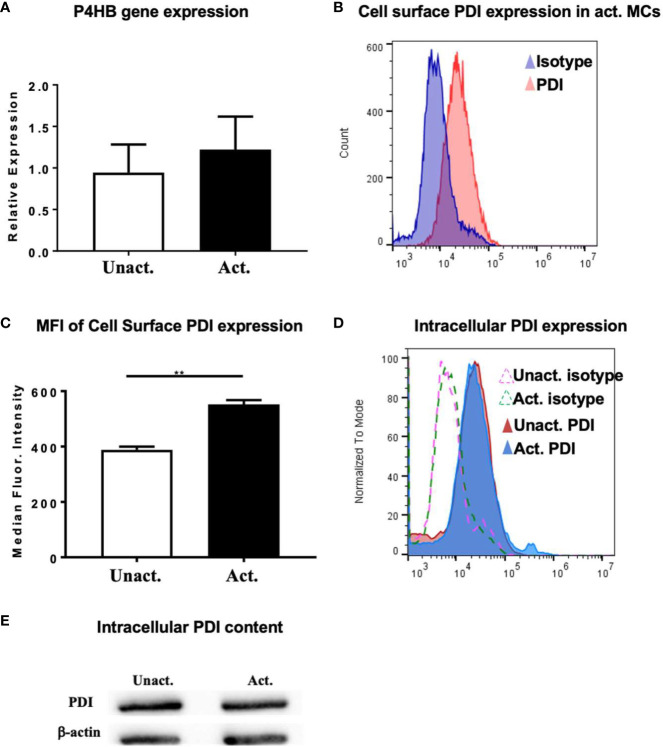
Upregulation of PDI expression in mast cells after activation with IgE and antigen. **(A)** BMMCs were activated with DNP-IgE and antigen and cells were collected for RT-PCR analysis after 4 h. Expression of P4HB is shown. **(B–D)** In other experiments, activated BMMCs or MC/9 cells and controls were examined for the expression of PDI family members by flow cytometry. **(B)** Histogram overlay depicting increase in PDI expression in activated BMMCs compared to isotype control is shown. **(C)** Plot depicting calculated median fluorescence intensity of PDI expression is shown. **(D)** Intracellular expression of PDI in fixed and permeabilized cells is shown. Isotype controls are represented as dashed lines. **(E)** Western blot for PDI in MC/9 cells. Data are representative of 2 independent experiments. **p < 0.01 (student’s t-test).

To further explore the role of PDI and evaluate its activity during mast cell activation, we assessed the expression of PDI in resting and IgE-activated BMMCs by quantitative PCR. PDI is the gene originally identified as the β-subunit of prolyl-4-hydroxylase (P4HB) that catalyzes the formation of 4-hydroxyproline in collagen (reviewed in (41)). We therefore assessed the expression of the P4HB gene in resting and activated mast cells. As seen in **Figure 5A**, basal expression of the P4HB gene may be observed in resting BMMCs consistent with its role as an intracellular chaperone. However, no further increase in gene expression was observed in IgE-activated BMMCs a few h after activation, suggesting that the intracellular transcriptional levels of PDI remain stable after activation has occurred (**Figure 5A**). This is not surprising as it is the increased enzymatic activity of PDI and not its cellular expression levels that contribute to changes in cellular function.

The secretion and extracellular activity of PDI in endothelial cells and platelets is well-established (16, 40, 42, 43), but its extracellular role in mast cells is unknown. To determine whether PDI can be detected on the surface of mast cells, we assessed the secretion of PDI using flow cytometry. As seen in **Figures 5B, C**, extracellular PDI can be detected on both resting and activated mast cells, with increased expression being observed a few min after activation. Quantification of the median fluorescence intensity of PDI expression revealed a significant increase in the presence of this molecule on the surfaces of cells activated with IgE and antigen (**Figure 5C**). These data therefore suggest that extracellular PDI is present on the cell membranes of resting mast cells, where it has the potential to modulate cellular function *via* its enzymatic activity. The secretion of extracellular PDI is further enhanced soon after IgE activation has occurred. Next, to examine the levels of intracellular PDI protein in mast cells, we evaluated its presence in unactivated and IgE-activated mast cells six h after IgE-activation had occurred. As observed in **Figure 5A** above, intracellular PDI was detected both constitutively and in activated mast cells by flow cytometry (**Figure 5D**). A small but demonstrable increase was observed in activated cells, although the overall levels appeared to be similar (**Figure 5D**), consistent with observations of intracellular PDI levels in other cell types (16, 36, 38–40). This was also confirmed by Western blot analysis (**Figure 5E**). These data therefore demonstrate that PDI is secreted on the surfaces of mast cells, where they may be a likely target of naturally occurring food-derived PDI inhibitors.

### PDI Inhibition During Mast Cell Activation Is Sufficient to Suppress IgE-Induced Cytokine Production

**Figure 6:**
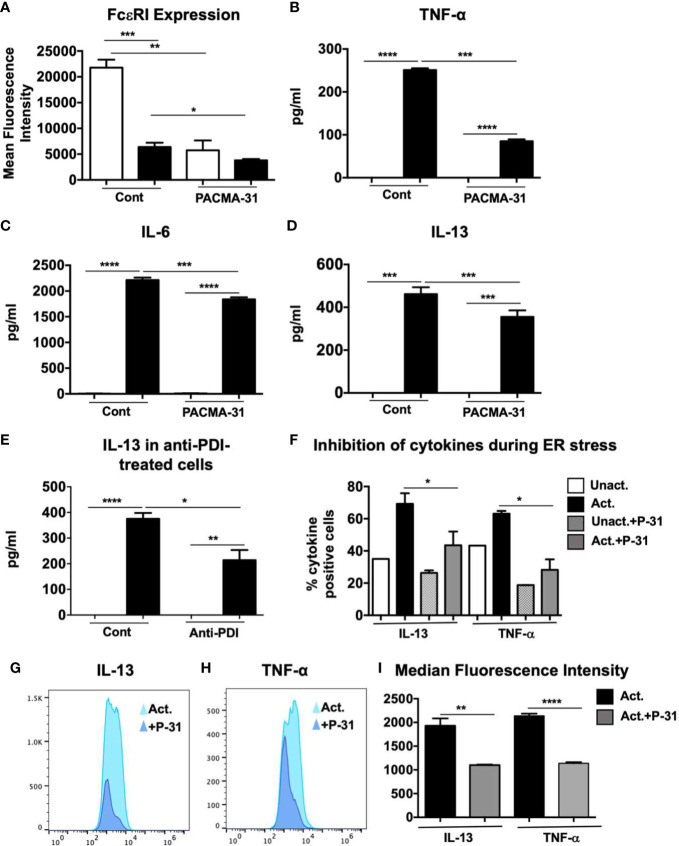
Evaluation of FcεRI expression, cell surface PDI inhibition and ER stress in PACMA-31–treated and IgE-activated BMMCs.** (A)** BMMCs were cultured with PACMA-31 overnight and the expression of Fc*ε*RI was assessed in unactivated and activated cells. Mean fluorescence intensity is shown. **(B–D)** BMMCs were treated with 30 μM PACMA-31 for 30 min and activated *via* IgE and antigen stimulation. Supernatants were collected 6 h after activation and ELISAs were performed for respective cytokines. **(E)** BMMCs were pre-treated with anti-PDI mAb for 6 h and activated *via* IgE and Ag. IL-13 secretion into cell supernatants was assessed by ELISA. **(F–I)** BMMCs were pre-treated with PACMA-31 overnight and activated *via* IgE and antigen. ER stress was induced by adding Brefeldin A during activation. Intracellular staining was performed 6 h later. **(F)** Numbers of cytokine producing cells **(G–H)** representative histogram overlays and **(I)** Median Fluorescence intensity for cytokines is shown. Data are representative of 2 independent experiments. *p < 0.05; **p < 0.005; ***p < 0.001; ****p < 0.0001 (student’s t-test).

The data above suggest that PDI can be secreted by mast cells after activation, with the potential to modulate downstream events such as the transcription of cytokine genes. To further assess the role of PDI inhibition on mast cell activation, we examined the effects of PACMA-31 pre-treatment on the expression of the high affinity receptor for IgE, Fc*ε*RI, on resting and activated BMMCs. As observed in **Figure 6A**, resting BMMCs express high levels of unbound Fc*ε*RI. In contrast, pre-incubation with PACMA-31 significantly decreased the expression of the receptor in BMMCs. In activated mast cells, the overall intensity of unbound Fc*ε*RI expression was decreased as would be expected as a consequence of saturation by IgE molecules. However, this was further reduced in PACMA-31-exposed and IgE-activated BMMCs.

It is possible that in the experiments above, the effects of PACMA-31 were mediated *via* inhibition of constitutively present cell surface PDI. To further investigate the effects of PDI inhibition during mast cell activation and to determine whether activation-associated PDI activity can modulate downstream cellular events, BMMCs were incubated with PACMA-31 starting 30 min prior to activation with DNP-BSA. Six h post-challenge with antigen, the levels of cytokines in supernatants were assessed. As observed in **Figures 6B–D**, the levels of TNF-α, IL-6, and IL-13 were suppressed in the PACMA-31–treated samples compared to untreated controls, suggesting that inhibition of activation-induced PDI activity may be sufficient to alter mast cell responses *in vivo*. Furthermore, inhibition of PDI was sufficient to attenuate the suppression of both pre-formed mediators such as TNF-α as well as *de novo* synthesized cytokines such as IL-13. To further confirm the effects of inhibition of cell surface PDI, BMMCs were treated with an anti-PDI mAb (Abcam), followed by activation *via* IgE and antigen (**Figure 6E**). As expected, extracellular PDI blockade also resulted in suppression of mast cell-derived IL-13 as seen in **Figure 6E**. Lastly, to determine whether PDI blockade by PACMA-31 during ER stress can inhibit the production of mast cell-derived cytokines, BMMCs were cultured with brefeldin A just before activating with DNP-BSA as described in **Figure 1**. Pre-treatment of cells with PACMA-31 significantly attenuated the capacity of BMMCs to produce IL-13 and TNF-α as shown in **Figures 6F to H**. Furthermore, the median fluorescence intensity of both cytokines was also decreased in PACMA-31-treated cells (**Figures 6G–I**). These data therefore strongly suggest that IgE-mediated mast cell activation can be regulated by the catalytic activity of mast cell surface PDI.

### PDI Inhibition Suppresses Mast Cell Responses in a Mouse Model of Food Allergy

**Figure 7:**
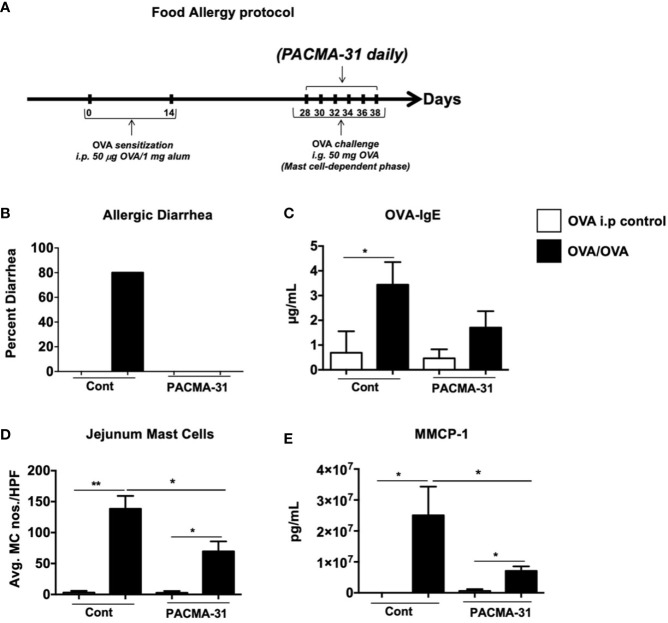
PACMA-31 treatment suppresses the development of food allergy in OVA-sensitized and challenged mice.** (A)** BALB/c mice were sensitized and challenged with OVA as shown. Some groups of animals were also gavaged with 300 µg PACMA-31 suspended in 1% CMC. **(B)** Percent of diarrhea-positive animals. **(C)** Serum OVA-IgE levels **(D)** Numbers of CAE^+^ jejunal mast cells. **(E)** Serum mMCP-1 levels are shown. n=7 mice/group. Data are representative of 2 independent experiments. *p < 0.05; **p < 0.01 (student’s t-test).

Our data above demonstrate a significant role for PDI during mast cell activation and function in cell culture. To ascertain whether blocking PDI activity *in vivo* will have similar effects on mast cell responses, we assessed the effects of PACMA-31 treatment in a mast cell-mediated model of intestinal food anaphylaxis. Briefly, mice were sensitized and challenged with the egg allergen OVA as previously described (24) (**Figure 7A**). Beginning one day prior to challenge with OVA, some groups of mice were orally gavaged with PACMA-31 suspended in 1% carboxy methyl cellulose as previously described (4) (**Figure 7A**). As observed in **Figure 7B**, OVA-sensitized and challenged mice developed profuse diarrhea in comparison with OVA-sensitized controls. This correlated with an increase in OVA-specific IgE production as enumerated in the serum (**Figure 7C**). In contrast, PACMA-31–gavaged animals did not develop diarrhea and exhibited decreased serum OVA-IgE levels (**Figures 7B, C**).

**Figure 8:**
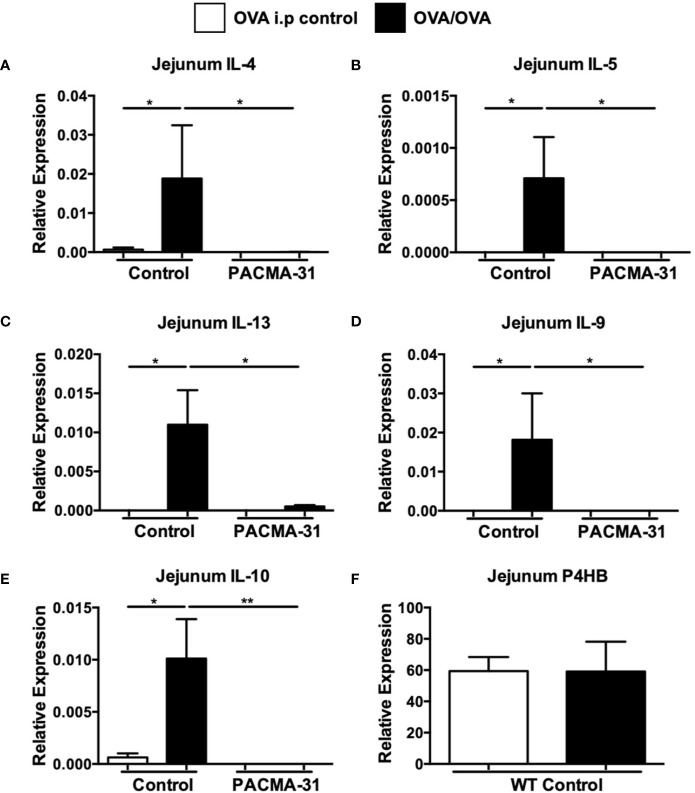
Expression of intestinal PDI in allergic mice and suppression of jejunal Th2 cytokine expression by PACMA-31. BALB/c mice were sensitized and challenged with OVA. Some groups of animals were also gavaged with 300 µg PACMA-31 suspended in 1% CMC. The expression of mRNA for various cytokines **(A–E)**, and **(F)** P4HB was assessed using established Taqman probes and RT-PCR. n=7 mice/group. Data are representative of 2 independent experiments. *p < 0.05; **p < 0.01 (student’s t-test).

Furthermore, enumeration of mast cells in the small intestine revealed a significant upregulation of chloroacetate esterase-positive mast cells in the jejunum of OVA-sensitized and challenged mice (**Figure 7D** and **Supplementary Figure 3**). Far fewer mast cells were observed in the jejunae of PACMA-31–treated animals (**Figure 7D** and **Supplementary Figure 3**). Similarly, the production of murine mast cell protease-1 (mMCP-1) was enhanced in OVA-challenged allergic mice compared to unchallenged controls (**Figure 7E**). In contrast, serum levels of this enzyme were decreased in PACMA-31–treated mice, suggesting decreased mast cell activation in these animals (**Figure 7E**). Furthermore, the expression of various T_H_2-type cytokines including IL-4, IL-5, IL-13, IL-9, and IL-10 was significantly reduced in the OVA-challenged PACMA-31-treated group compared to the OVA-challenged controls (**Figures 8A–E**).

The data above suggest that PDI activity *in vivo* may be enhanced during allergic responses and its inhibition can modulate mast cell function during food allergy. To determine whether this correlated with increased expression of PDI family members in the intestines of allergic mice, we assessed the levels of P4HB in the jejunae of experimental animals. As expected, expression of these genes was observed in the intestines of both controls and allergic animals and unchanged, consistent with enzymatic inhibition as opposed to decreased expression (**Figure 8F**).

**Figure 9:**
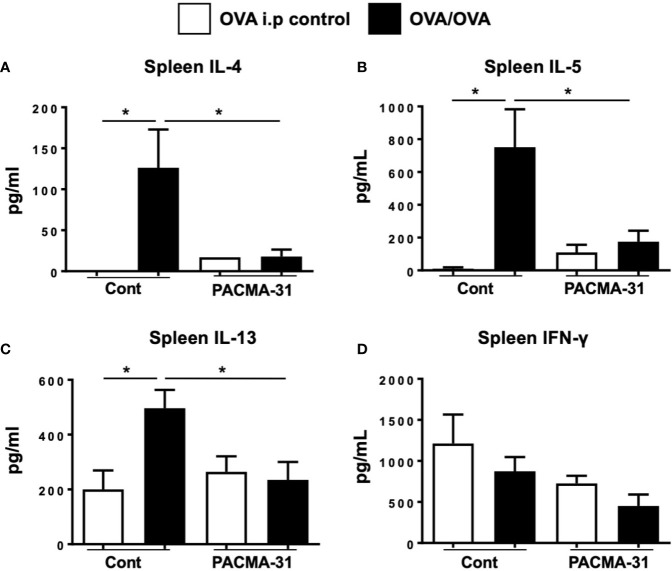
Impaired T_H_2 cytokine production by spleen cells from OVA-challenged, PACMA-31–treated mice. BALB/c mice were sensitized and challenged with OVA. Some groups of animals were also gavaged with 300 µg PACMA-31 suspended in 1% CMC. Spleen cells were stimulated with OVA for 72 h. Levels of the cytokines **(A)** IL-4, **(B)** IL-5, **(C)** IL-13, and **(D)** IFN-γ were enumerated in the supernatants by ELISA. n=4 mice/group. Data are representative of 2 independent experiments. *p < 0.05 (student’s t-test).

Lastly, the effects of PDI inhibition on systemic T_H_2 cytokine production were assessed by stimulating spleen cells with OVA and examining the production of cytokines. As expected, the production of IL-4, IL-5, and IL-13 (**Figures 9A–C**) was enhanced in OVA-stimulated splenic cultures compared to unstimulated controls. In contrast, no enhancement of cytokine production was seen in similarly stimulated cells from PACMA-31–gavaged animals. Also, no differences in IFN-γ production were observed across all groups examined (**Figure 9D**). A similar pattern was observed in cells polyclonally stimulated with T cell agonists (**Supplementary Figure 4**). These data therefore suggest that PDI blockade can suppress T_H_2-specific cytokine production during allergic inflammation.

## Discussion

In this study, we report for the first time an important role for PDI in the modulation of mast cell homeostasis and mast cell-mediated allergic responses. Collectively, our data demonstrate that mast cells express PDI both constitutively and during IgE-mediated activation. The presence of PDI was also detected in the intestines of enterally-challenged allergic mice. Blockade of PDI activity at both the cellular and physiological levels resulted in a profound suppression of mast cell-mediated responses including decreased mast cell proliferative capacity, reduced secretion of mast cell cytokines, and protection from the development of food allergy.

PDI, a multifunctional ER thiol isomerase, is a 55-kDa protein that is the prototype of the PDI family of proteins. The PDI family comprises of 21 members (44). PDI family members play a critical role in the regulation of protein folding and assembly, both during physiological homeostasis as well as in conditions of cellular stress (15). Increased generation of unfolded or misfolded proteins during ER stress activates the UPR, resulting in reduced protein synthesis, an increase in the ER curvature, and the activation of PDI and other chaperones (45, 46).

The development of inflammation is thought to activate the UPR, resulting in the enhancement of protein synthesis and folding, and consequently increased PDI activity (47). Complex allergens in particular, such as food-derived proteins, have the potential to induce ER stress and upregulate the function of PDI family members (48). Despite this, the effects of PDI on immune cells has been poorly studied, and very little is known regarding its functions during allergic inflammation. Interestingly, a recent study demonstrated a critical role for a PDI family member, ERp57 in airway allergic responses (49). The expression of ERp57 was increased in lung epithelial cells in both allergen-challenged patients and in mice. Deletion of ERp57 in house dust-mite-challenged mice resulted in decreased airway inflammation and hyperreactivity, accompanied by decreased disulfide bridges in eotaxin, epidermal growth factor, and periostin in the lungs of allergic animals. These data suggest that modulation of PDI activity *in vivo* can influence the outcome of allergic sensitization and challenge.

We hypothesized that food-derived substances may have the potential to modulate mast cell responses during food allergy by inhibiting the activity of PDI. We have previously shown that widely consumed dietary components such as curcumin can suppress mast cell activation and inhibit the development of food allergy in mice (4). The suppression of mast cells by curcumin in this model was dependent on the inhibition of NF-κB activation (4). Similarly, Lee et al. also described anti-allergic effects of curcumin on mast cells (7). They found that the inhibitory effects of curcumin on cultured mast cells were mediated *via* inhibition of Syk kinase activity. In this study, we report the PDI inhibitory activity of curcumin, suggesting another potential mechanism for its anti-allergic effects. Since PDI is involved in the formation of disulfide bonds which is critical for the folding of many proteins including transcription factors, it is likely that the previously described suppression of proteins by curcumin and other dietary substances occurs *via* inhibition of PDI catalytic activity. In this context, in addition to curcumin, we demonstrate that another well-known dietary PDI modulator, rutin, also inhibits mast cell activation in our model. This is consistent with reported observations where quercetin and its derivatives, have been demonstrated to have potent anti-PDI activity *in vivo* in various clinical trials (27, 28). In this context, our data suggest that anti-inflammatory compounds such as curcumin and the flavonoids, which are ubiquitous in various types of diets including fruits, vegetables, wines and teas, also have the potential to alter PDI activity in human mast cells and suppress the development of allergic inflammation (27, 28, 50). While we did not specifically examine human mast cells or PDI expression in these cells in this study, it will be important to determine whether PDI has similar effects on IgE-mediated activation in human cells. In this context, interestingly, quercetin and other polyphenols have been shown to modulate human mast cell activity in other studies (51, 52).

Our data suggests that allergen-induced mast cell activation may be a likely target of PDI-dependent modulation by dietary substances, thereby mitigating the overall magnitude of the allergic response. Dietary PDI modulators may possibly attenuate the activity of extracellular or cell surface PDI on mast cells and other immune cells, thereby suppressing their overall activation, and decreasing the production of proinflammatory mediators. This was evidenced by treatment with various PDI inhibitors, which suppressed the IgE-mediated activation of mast cells and inhibited their degranulation and cytokine secretion. Furthermore, treatment with PACMA-31, an orally bioactive, irreversible inhibitor of PDI suppressed mast cell responses both in cell culture and during the development of food allergy, suggesting that therapeutic targeting of PDI in allergic patients may prove to be of benefit. Interestingly, PDI inhibition also attenuated the long-term survival of BMMCs, suggesting that modulation of PDI activity *in vivo* can influence the homeostasis of mast cells. Nonetheless, the effects of PDI inhibition on mast cell degranulation and cytokine secretion were unrelated to its effects on cell viability, as no significant effects on cellular viability were observed within the first 48 h, *i.e.* the percentages of live and dead cells between untreated and treated groups were comparable at this time point (**Figures 3C** and **4F**).

In this report, we demonstrate for the first time the presence of PDI molecules on the surface of mast cells. Here, their enhanced extracellular activity (which can be blocked by PDI inhibitors) during mast cell activation may modulate downstream cellular events, contributing to changes in molecular and cellular function. In this context, cell-surface specific PDI has been found to be important for the functions of hepatocytes, endothelial cells, and platelets (27, 53). Furthermore, PDI has also been shown to be secreted into cell culture supernatants, which we did not ascertain in these studies (reviewed in (15)). It is thought that in these locations, PDI assists with redox protein folding, intramolecular thiol-disulfide exchanges and isomerization activities, as a result of highly specific interactions with various substrates. Future studies aimed at dissecting the contributions of both extracellular and intracellular PDI to mast cell activation will shed further light on the mechanisms by which PDI enhances mast cell responses. In particular, the effects of PDI catalytic activity on the formation of disulfide bonds in various proteins produced by mast cells or that activate them will be important to characterize. In this context, recent studies have elucidated novel roles for the disulfide dimer histamine-releasing factor (HRF) in mast cell-mediated allergic responses (54, 55). HRF forms dimers which can cross-link with IgE on basophils and mast cells and induce the secretion of enhanced levels of histamine, IL-4 and IL-13. Both increased levels of HRF-reactive IgE as well as increased numbers of HRF dimers were found in a recently described study of mast cell-mediated food allergy (54, 55). Similarly, patients with food allergy also had increased levels of serum HRF-reactive IgE and blockade of HRF function in the mouse model as well as oral immunotherapy in patients inhibited the allergic response (54, 55). In light of our data above, it is possible that the generation of HRF dimers *in vivo* may depend on PDI activity, resulting in the enhanced secretion of these proteins during allergic responses and the amplification of mast cell-mediated reactions.

The effects of PDI may also extend beyond its known isomerization activities. For example, recent studies demonstrate that PDI can increase the levels of reactive oxygen species (56) thus directly inducing oxidative stress and apoptosis, as well as activate transcription factors, such as NF- κB and AP-1 (57). In this context, we have previously demonstrated that the protective effects of curcumin on mast cells are mediated *via* inhibition of NF-κB activation (4), whereas another study demonstrated that flavonoids such as rutin can inhibit reactive oxygen species in mast cells (51). As such, further analysis of the effects of PDI inhibition on these and other parameters in mast cells will help elucidate the roles of PDI in these areas.

Lastly, this study demonstrates that PDI blockade has a profound effect on mast cell homeostasis (intestinal mast cell numbers), activation (mMCP-1 levels), and mast cell-mediated effects such as allergic diarrhea in the IgE and mast cell-dependent model of OVA-induced food allergy. However, although we have not examined other immune cell types in this study, it is likely that the activity of PDI family members is also altered in other cells during allergic responses. Particularly, our data in **Figure 9** demonstrate that OVA-specific splenic responses are also suppressed in the food allergy model, thereby implicating a role for PDI in the modulation of allergen-specific T cells. Future studies aimed at examining the effects of PDI on various immune cells during food allergy are therefore necessary to clarify the differential effects of PDI modulation on immune cells and their contribution to the development of food allergy.

Taken together, our data suggest that PDI and its related family members may play vital roles in the regulation of mast cell activation during the allergic response, and that *in vivo* blockade of their activity may prove to be of therapeutic benefit in patients with mast cell-mediated disorders.

## Data Availability Statement

The original contributions presented in the study are included in the article/**Supplementary Material**. Further inquiries can be directed to the corresponding author.

## Ethics Statement

The animal study was reviewed and approved by The Institutional Animal Care and Use Committee at Western New England University.

## Author Contributions

CBM and DRK conceived and directed the project. DK, SP, JG, JR, EK, CG, MP, NS, DRK, and CBM performed experiments and analyzed data. DK, SP, JG, DRK, SSS and CBM prepared figures for the manuscript. CBM, DK and DRK wrote the paper. All authors contributed to the article and approved the submitted version.

## Funding

This project was supported by funds from the National Institutes of Health grants: NIAID R15AI107668 (CM) and NCI R21CA231000 (DRK).

## Conflict of Interest

CBM and DRK are authors on a patent application submitted by Western New England University regarding the role of thiol isomerases in mast cell activation.

The remaining authors declare that the research was conducted in the absence of any commercial or financial relationships that could be construed as a potential conflict of interest.

## Supplementary Material

Supplementary Figure 1
*Pre-treatment with bacitracin and rutin suppresses IgE-induced BMMC cytokine gene expression.* BMMCs were treated with increasing doses of bacitracin (Bac) **(A–C)** or rutin (rut) **(D–F)** and activated *via* IgE and antigen stimulation. Cells were pooled and collected 1 h after activation and RNA and cDNA were prepared using established protocols. qRT-PCR was performed using Taqman probes. Expression of genes was calculated relative to that of GAPDH.

Supplementary Figure 2
*Comparison of the percent PDI inhibitory activity of curcumin and PACMA-31.* The PDI inhibitory activity of curcumin and PACMA-31 were evaluated using the insulin turbidimetric assay. Bar graphs representing inhibition at the 40 min time point for various doses of the inhibitors are shown. Significance at each dose was calculated against the PDI added and untreated control group using one-way ANOVA (p< p=<0.0001).

Supplementary Figure 3
*Histology of small intestine from OVA-sensitized and challenged and PACMA-31*–*treated mice.* Food allergy was induced in mice by sensitizing and challenging with OVA as in **Figure 7A**. Some groups were treated with PACMA-31 orally. Representative histological images depicting CAE-positive mast cells is shown for the **(A)** WT control **(B)** WT OVA **(C)** P-31 control and **(D, E)** two P-31 OVA groups. Mast cells are shown by an arrow.

Supplementary Figure 4
*Impaired polyclonal cytokine production by spleen cells from OVA-challenged, PACMA-31*–*treated mice.* BALB/c mice were sensitized and challenged with OVA to induce food allergy. Some groups of animals were also gavaged with 300 µg PACMA-31 suspended in 1% CMC. Upon sacrifice, spleen cells were stimulated with anti-CD3 and anti-CD28 for 72 h. Levels of the cytokines **(A)** IL-4 **(B)** IL-5 **(C)** IL-13, and **(D)** IFN-γ were enumerated in the supernatants by ELISA. n = 4–7 mice/group. Data are representative of 2 independent experiments. *=p<0.05; **=p<0.01; ***=p<0.0001 (student’s t-test).

## Abbreviations

OVA, ovalbumin; WT, wild-type; BMMCs, bone marrow-derived mast cells; PACMA-31, propynoic acid carbamoyl methyl amide; Rutin, quercetin-3-rutinoside.
