# Supplementary figures and images for: Protein Disulfide Isomerases Regulate IgE-Mediated Mast Cell Responses and Their Inhibition Confers Protective Effects During Food Allergy
Source: Front Immunol. 2020 Dec 22;11:606837. doi: 10.3389/fimmu.2020.606837 (PMC7783394; doi:10.3389/fimmu.2020.606837)

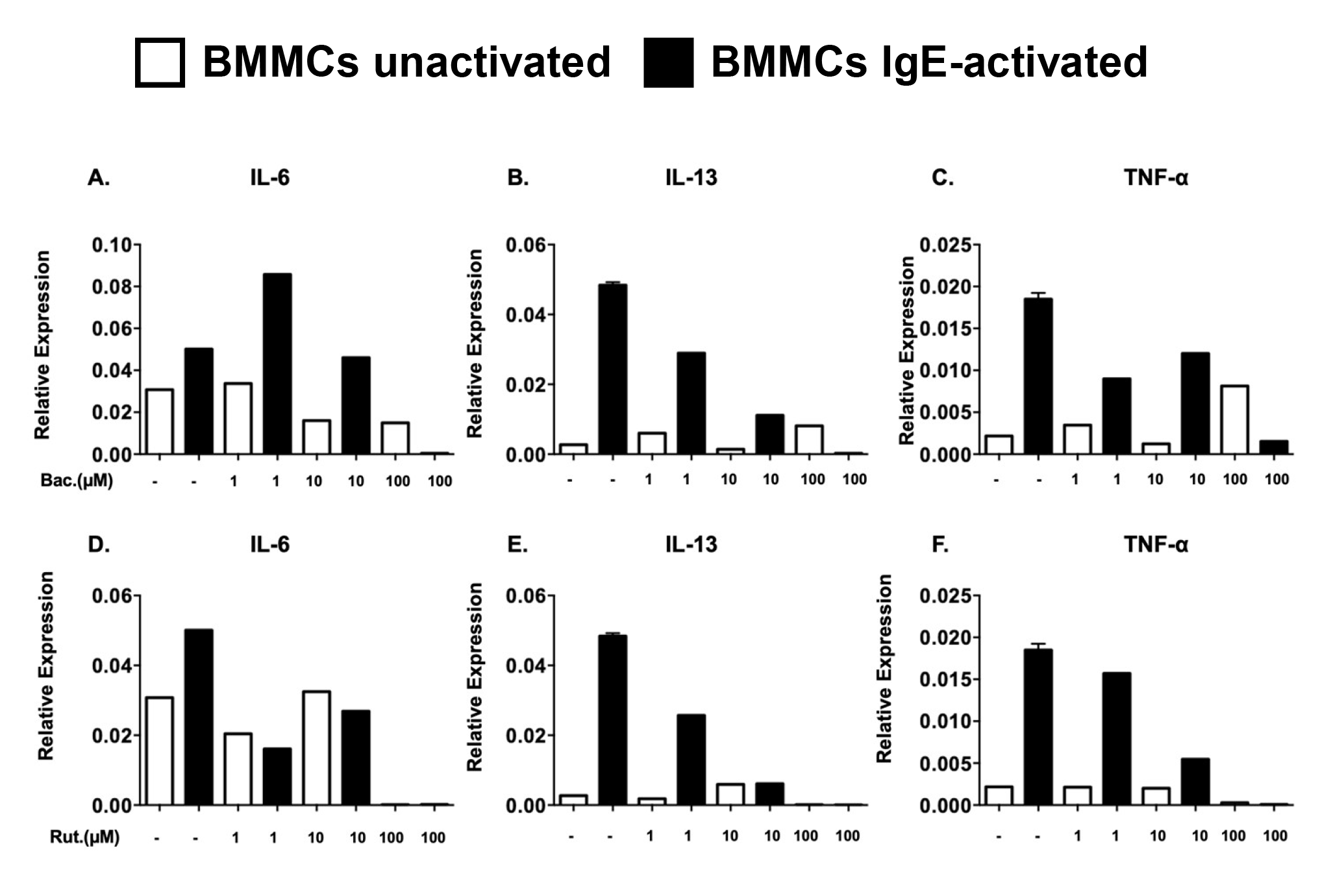

Supplement: Supplementary Figure 1 — Pre-treatment with bacitracin and rutin suppresses IgE-induced BMMC cytokine gene expression. BMMCs were treated with increasing doses of bacitracin (Bac) (A–C) or rutin (rut) (D–F) and activated via IgE and antigen stimulation. Cells were pooled and collected 1 h after activation and RNA and cDNA were prepared using established protocols. qRT-PCR was performed using Taqman probes. Expression of genes was calculated relative to that of GAPDH. [file Image_1.tif]

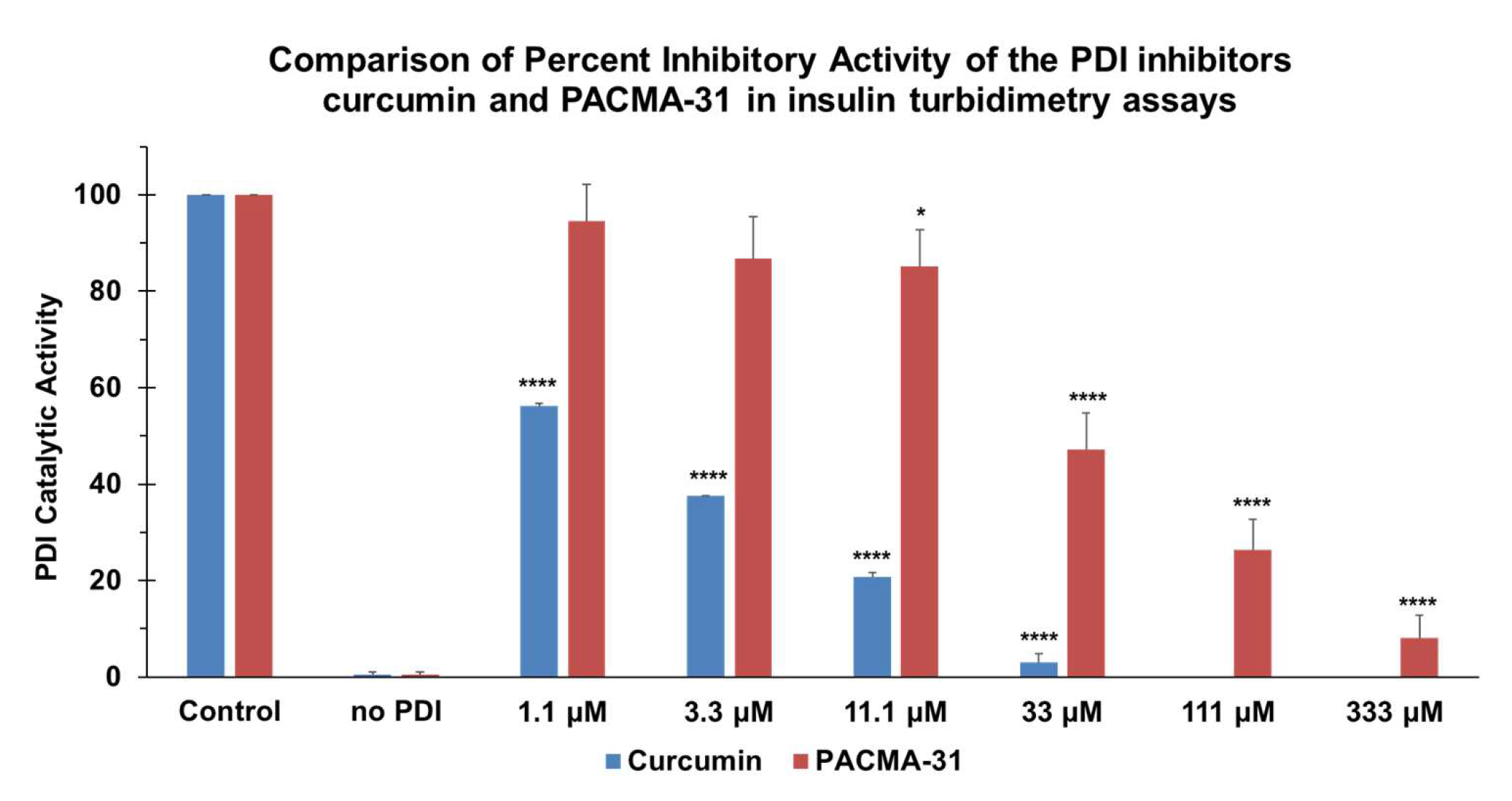

Supplement: Supplementary Figure 2 — Comparison of the percent PDI inhibitory activity of curcumin and PACMA-31. The PDI inhibitory activity of curcumin and PACMA-31 were evaluated using the insulin turbidimetric assay. Bar graphs representing inhibition at the 40 min time point for various doses of the inhibitors are shown. Significance at each dose was calculated against the PDI added and untreated control group using one-way ANOVA (p< p=<0.0001). [file Image_2.tif]

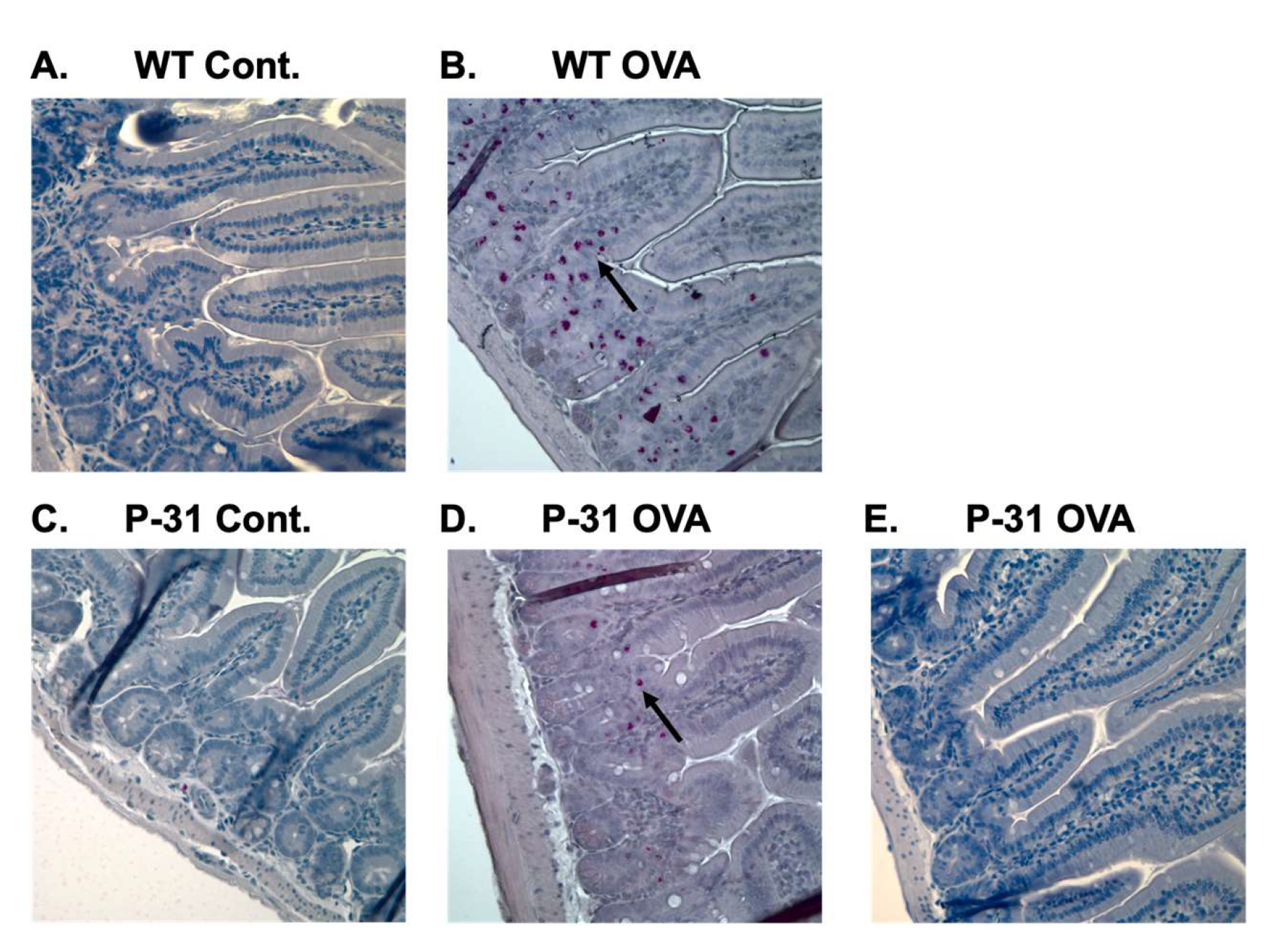

Supplement: Supplementary Figure 3 — Histology of small intestine from OVA-sensitized and challenged and PACMA-31–treated mice. Food allergy was induced in mice by sensitizing and challenging with OVA as in Figure 7A . Some groups were treated with PACMA-31 orally. Representative histological images depicting CAE-positive mast cells is shown for the (A) WT control (B) WT OVA (C) P-31 control and (D, E) two P-31 OVA groups. Mast cells are shown by an arrow. [file Image_3.tif]

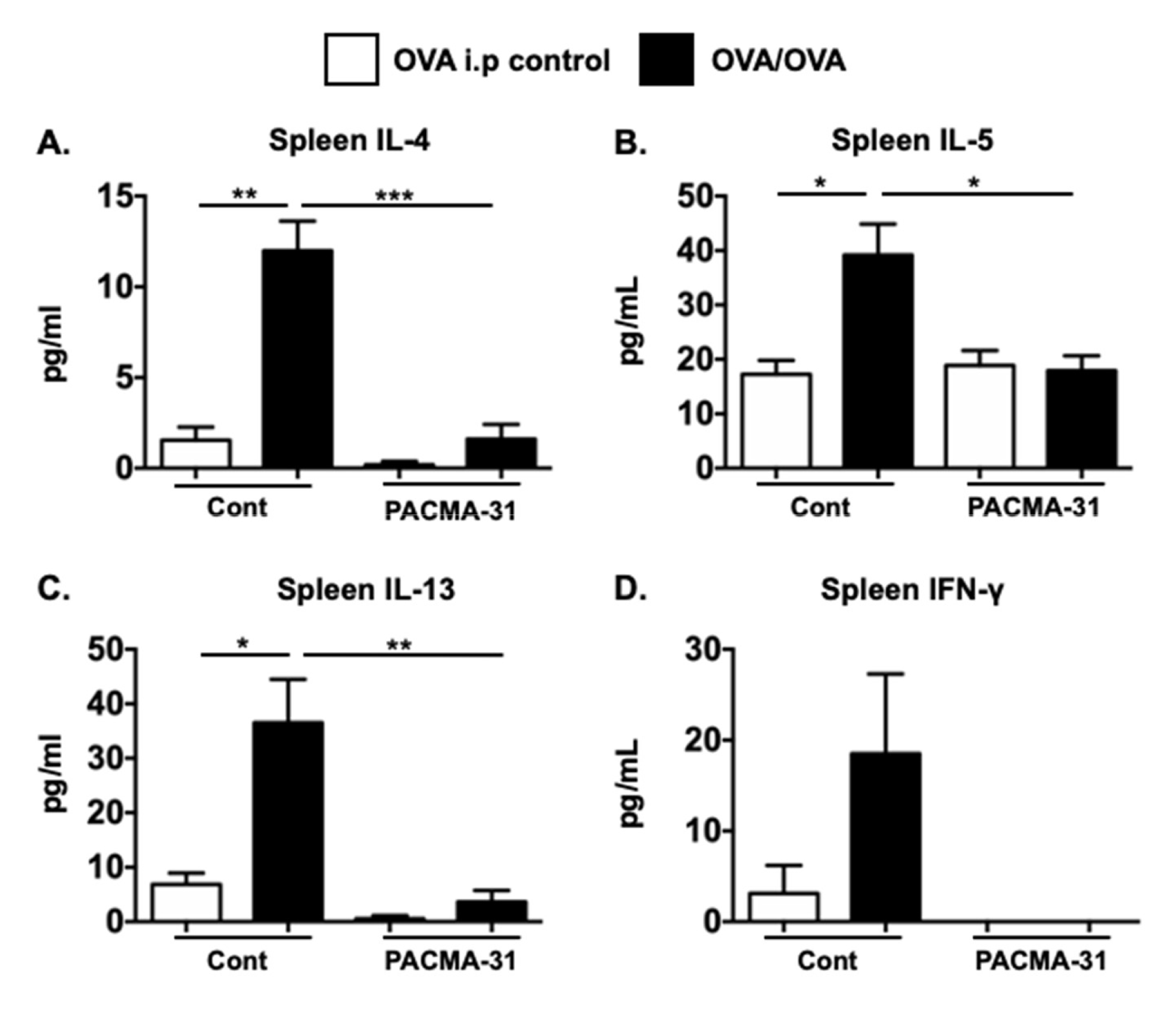

Supplement: Supplementary Figure 4 — Impaired polyclonal cytokine production by spleen cells from OVA-challenged, PACMA-31–treated mice. BALB/c mice were sensitized and challenged with OVA to induce food allergy. Some groups of animals were also gavaged with 300 µg PACMA-31 suspended in 1% CMC. Upon sacrifice, spleen cells were stimulated with anti-CD3 and anti-CD28 for 72 h. Levels of the cytokines (A) IL-4 (B) IL-5 (C) IL-13, and (D) IFN-γ were enumerated in the supernatants by ELISA. n = 4–7 mice/group. Data are representative of 2 independent experiments. *=p<0.05; **=p<0.01; ***=p<0.0001 (student’s t-test). [file Image_4.tif]
